# Social frames promoting sharing in Norwegian Recovery Colleges: an ethnographic study

**DOI:** 10.3389/fpsyt.2025.1606180

**Published:** 2025-06-23

**Authors:** Therese Ersvær Sjursæther, Christine Øye, Sonja Mellingen

**Affiliations:** ^1^ Western Norway University of Applied Sciences, Faculty of Health and Social Science, Bergen, Norway; ^2^ Western Norway University of Applied Sciences, Faculty of Health and Social Science, Stord, Norway

**Keywords:** Recovery College, mental health service, substance use and mental health challenges, social interaction, sharing and disclosing, co-creation, adult learning

## Abstract

**Introduction:**

Recovery Colleges (RCs) for people with substance use and mental health challenges represent an innovation in mental health services, emphasising co-creation and adult learning. Students and course facilitators with diverse experiences engage in collaborative learning in these settings by sharing experiences, knowledge, and skills. This paper examines the social frameworks that facilitate or hinder sharing within RCs.

**Methods:**

We conducted an ethnographic study in two distinct RC settings, using participatory observation and semi-structured interviews with facilitators, students, and leaders. We employed Goffman’s frame analysis to understand the social framework in RC and how its organisational structures and physical premises influence sharing among students and facilitators.

**Results:**

Our results reveal layers of social frameworks that emphasise learning, recovery, strengths, equality, and open discussions about mental health. Organisational structures and physical premises significantly support or hinder these social frameworks. Clear communication, preparatory conversations, respect for boundaries, and neutral settings were identified as key aspects promoting sharing. Conversely, focus on diagnoses, top-down attitudes, inadequate preparations, excessive facilitator involvement, health-related settings, and overly exposed arrangements could inhibit sharing.

**Discussion:**

The organic interactions within RC courses create complexities in understanding the promoters and inhibitors of sharing within these social frameworks. What promotes sharing in one setting can inhibit sharing in another. We illustrated situations where disruptions to the frames either promoted active participation for some or inhibited sharing for those who felt overwhelmed. Recognising this complexity is crucial for facilitators in RCs to effectively frame sharing and achieve mutual learning among students.

## 1 Introduction

The first Norwegian Recovery Colleges (RCs) were launched in 2019 for individuals facing substance use and mental health challenges, their families, and mental health practitioners, inspired by successful models in England (1). Since then, seven RCs have been established across various municipalities, significantly expanding this approach. RCs represent an innovative shift in addressing mental health and substance use challenges by reframing the traditional recovery-from-illness model to one rooted in learning principles (1, 2). Reframing (3) involves a new understanding and approach to substance use and mental health challenges, enabling service users to assume new social roles as students (4).

RCs are perceived as more inclusive and empowering than conventional educational settings, as they maintain a recovery-focused context while transitioning from a therapeutic to an academic model. By embracing the philosophy of personal and social recovery, RCs complement conventional mental health services by promoting opportunities to discover new ways to live fulfilling lives despite their challenges (2). Through participating in RC courses, students gain self-awareness, understand their difficulties, and develop practical self-management skills (5). They can choose from a selection of courses offered by the RCs, selecting those they consider most suitable for their needs (1).

High-fidelity criteria derived from research serve as guiding principles for RC operations (1, 2, 5). The RECOLLECT checklist is the latest refined and expanded version (5). Key principles, such as *valuing equality, co-creation* and *adult learning* (5, 6) ensure that RCs harness diverse experiences and resources from diverse actors (7, 8), including individuals with lived experience of mental health challenges and those with formal mental health training. Facilitators from both backgrounds co-design and co-deliver all aspects of the program (1, 5), aiming to minimise power imbalances between professional and experiential knowledge (9).

The diversity among students and facilitators ensures a rich exchange of perspectives, fostering co-creation of knowledge and mutual learning. Facilitators with experiential knowledge and formal mental health training are expected to be open to personal change and growth, and to participate in the sharing practice during courses (9–11). Through structured activities and sharing experiences, knowledge, and skills, students are encouraged to challenge their perspectives and explore new possibilities, facilitating personal growth and development. They learn from other students and facilitators with diverse backgrounds, and collaborative interactions among themselves (9). As students gain access to knowledge, they also support and guide other students and facilitators, reinforcing the mutual learning process (12). This dynamic creates an environment where everyone can gain access to valuable tools and resources to manage their challenges (6, 13–15).

Learning and mutual support within groups foster *social connectedness* and student relationships that often extend beyond the classroom (1). RC courses should adhere to *recovery* principles and focus on *community* by assisting students in integrating into community roles and activities (5). These courses should be *inclusive and accessible to all* citizens with minimal restrictions (2, 5), offering *distinct content tailored to meet students’ needs* (5). The RCs should adopt *a strengths-based, progressive approach* that prioritises strengths over problems and emphasises the process of being and becoming (2, 5). The location of the RCs may be independent or linked with other services (5).

Previous studies indicate that students emphasise co-leadership and support from facilitators with lived experience in promoting effective learning. The learning mechanism they underscore involves learning within a diverse group of students and acquiring new knowledge (12). Further, the emphasis is on voluntary participation, relevant content and practical tools (14). Facilitators’ experiences cultivate feelings of empathy and reduce stigma by sharing their mental health experiences (16), which fosters relational bonds and provides students with broader perspectives (10). However, facilitators must balance their contributions to avoid discomfort and ensure student engagement (17). Although the softening of roles is the most commonly reported change (4), this transition can be challenging for facilitators with formal mental health training (16).

Jones et al. (18) suggest that RC operations are inherently idiosyncratic and adapted to local contexts. Institutional contexts, positioning, and interpretations of key terms influence daily operations and stakeholder involvement. This means that different RCs have different social frameworks, including organisational and physical aspects of the frameworks, that play a crucial role in shaping the experiences of both students and facilitators. These frameworks can influence how students engage with the courses and interact with one another. Various factors that facilitate or hinder the implementation of RCs and contribute to student drop-outs have been identified, noting that promoters in one setting can function as inhibitors in another. Practical obstacles, distressing interactions with facilitators or peer students, and course-related issues, such as unclear expectations, are notable barriers (6, 19). Clear communication has been highlighted to mitigate these barriers and promote attendance (6).

Despite the extensive literature on RCs, the impact of social frameworks on students’ engagement in sharing remains underexplored. This paper, employing Goffman’s frame analysis, which has previously been applied to a limited extent to data from RCs, aims to provide a micro-sociological understanding of the reframing from therapy to learning principles. Specifically, we explore social frameworks and describe how organisational and physical aspects influence sharing among students and facilitators. Therefore, our research is structured around the question: *How do social frames promote and inhibit sharing in Norwegian Recovery Colleges?*

## 2 Materials and methods

### 2.1 Research design

We conducted an ethnographic study (20) to explore the social frameworks within RCs at two distinct sites. Our methodology included participatory observations, focus groups, and semi-structured interviews, which enabled us to gather rich and detailed insights into how these frameworks operate at each site. Utilising two sites served as an analytical tool to identify and understand the unique characteristics and dynamics of the social frameworks. Instead of making consistently explicit comparisons, we emphasised what was prevalent across both sites and the most significant differences between them, as these findings may inform the generalisation to other settings.

### 2.2 Study setting and participants

The Norwegian RCs in this study hosted the courses at two locations, Site 1 and Site 2.

Site 1 is part of a competence centre within the health sector covering several municipalities, half of which run recovery courses. We primarily focused on the most established RC in a medium-sized municipality. The centre employs project-specific staff, while municipal facilitators formally engage in other municipal services. The two recovery courses they offer focus on collaboration between students and facilitators to identify personal goals inspired by the CHIME framework (21). Each course consists of 12 sessions, held twice weekly, with a capacity of 10 students.

Site 2 serves a larger region of a few hundred thousand residents. The staff includes two project managers, one with formal mental health training, the other with experiential knowledge of substance use and mental health challenges, and a part-time employee with similar experiential knowledge. Local municipality services support the program through staff for course development and facilitation. The first author participated in a five-week course on building healthy relationships and community. Other courses cover a variety of themes, including identity, life skills, art, music, and photography.

The study participants included course facilitators, leaders, and students from both locations. Facilitators and leaders with formal mental health training had diverse backgrounds in health and social education. Facilitators with experiential knowledge possessed personal experience with substance use and mental health challenges or had supported relatives facing such challenges. Additionally, facilitators and leaders took on the roles of course developers. Due to anonymity considerations, we referred to all these roles as “facilitator.” Although the RC is open to relatives and mental health service providers, the courses we examined were attended exclusively by students with lived experience related to substance use and/or mental health challenges, some of whom served as peer workers.

### 2.3 Data collection

We gathered data in two distinct periods. In autumn 2022, this included participatory observation and interviews with course facilitators and students at both sites. Participatory observation involved participating in two courses, including all the course activities, and sharing reflections with other students. We followed six facilitators and 14 students through participatory observations over 12 days and interviewed five of the facilitators and eight students. Additionally, we attended ten meetings on Microsoft Teams, where one specific course was co-created. The participatory observation method helped us develop an insider’s perspective, gain trust, and communicate closely with facilitators and students (20). The insights from the observations were instrumental in developing the interview guide for subsequent focus groups and semi-structured interviews.

We invited all students enrolled in the observed courses to participate in focus groups to discuss their experiences with co-creation, user involvement, sharing culture, and personal gains. Course facilitators assisted in arranging the focus groups towards the end of the recovery courses, which also served as a positive closure. Focus groups effectively provide information about experiences, attitudes, and views in an interactive environment (22).

After the courses, we conducted one focus group interview and two semi-structured interviews with facilitators from the two sites who consented to participate. The semi-structured interviews involved facilitators who did not participate in the focus group, providing rich, detailed narratives that complemented the broader themes identified during the focus group sessions (23).

The second period, in autumn 2024, was undertaken to gather additional data for this article, including 14 facilitators, three leaders and four students from the first round of data collection. We primarily focused on individuals who had developed and led the RCs and their courses. Additionally, we interviewed four students from the first round of interviews, focusing on their expectations, security factors, and the role of facilitators and students. Most of the interviews conducted during this period were held on Microsoft Teams.

Field notes and interviews were supplemented with documents, reports, and brochures from the RCs to enhance contextual understanding.

**Table 1** provides a detailed overview of the data collection.

**Table 1:** Data material.

Type of Data	Details	Duration	Study participants
Participatory Observation course delivery	Notes	12 days	3 facilitators of professional knowledge 3 facilitators of experiential knowledge 14 students with lived experience
Participatory observation course design	Notes	10 days	2 facilitators of professional knowledge 2 facilitators of experiential knowledge
Focus group interviews with RC leaders and facilitators (responsible for designing and delivering courses)	2 groups	2 hours	5 Facilitators with professional knowledge 2 Facilitators with experiential knowledge
Semi-structured interviews with RC leaders and facilitators (responsible for designing and delivering courses)	10 interviews	1-1.5 hours	8 Facilitators with professional knowledge 6 Facilitators with experiential knowledge
Semi-structured interviews with students	4 interviews	0.5–1 hour	4 students with lived experience
Documents about RC	Course material Four-year report Quality Requirements Brochures		

### 2.4 Ethics considerations

All procedures were conducted in accordance with the ethical guidelines outlined in the Declaration of Helsinki. SIKT - Norwegian Agency for Shared Services in Education and Research, recommended the study in 2022, project number 994561. In the context of ethical conduct, each RC’s leaders collectively consented to the participatory observation research activities. The researchers ensured that we followed all ethical guidelines, ensuring confidentiality and respecting the autonomy of all students. Anonymity was maintained by not distinguishing between the different sites. The ethical oversight process also involved the researcher’s self-disclosure at the first gathering, promoting transparency about the study’s purpose and the researcher’s role. We provided all facilitators and students in the course with detailed information about the focus groups one week before the scheduled interviews. Facilitators and students who participated in the interviews provided informed consent after being informed about the study’s purpose, procedures, potential risks, and their rights as study participants. We conducted focus groups on the last days the researcher participated in the course to safeguard those individuals who declined to participate in the interview.

### 2.5 Positionality of researchers

The quality of scientific knowledge and ethical decisions in qualitative research depends on the researcher’s role and integrity (23). The first author’s background as a social worker in Norwegian mental health services likely influenced the research process. Her interest in sharing practices stems from her experience in these services and her research on peer workers’ recommendations. She also has experience in facilitating psychoeducational trauma groups and tutoring mental health service providers, which informed her interest in participant contributions. To mitigate potential biases, the researcher maintained critical self-awareness, reflected on personal biases, and engaged in frequent supervision sessions to enhance the objectivity and credibility of the findings (20). Despite these measures, the potential influence of her background and experiences on the research process and findings cannot be eliminated.

### 2.6 Analytical framework

To capture the social frameworks in RCs and understand their dynamics, we turn to Goffman’s frame analysis, a method to study how people organise and understand social events and their subjective experiences [(3), pp. 10–11]. Goffman’s frame analysis explains how people organise their experiences and define situations using frameworks that govern social events and our subjective involvement. This article focuses on social frameworks, which provide a background for understanding events like RC courses, driven by human will, aim, and control. Social frameworks comprise several sub-frameworks, which we refer to as frames, that help us define the situation, determine acceptable behaviour, and organise involvement [(3), pp. 8–11]. The term frame refers to the background, setting, or context. It suggests that what happens during social interactions is influenced by unstated rules and principles, dictated by the overall situation [(3), p. xiii].

The function of social frameworks, known as guided doings, encompasses physical management and the social world, including social interactions, conflicts, and the various roles that emerge from these events. Participants gain a sense of what is happening and can become spontaneously engaged, with involvement expectations varying depending on the framework [(3), pp. 22–26]. The term keys indicates how a particular activity or interaction should be interpreted. Keyings refer to using these keys to reframe an activity or situation [(3), p. 44].

Frameworks are vulnerable to different participant interpretations. One individual might have an incorrect understanding, meaning they are misguided, out of touch, or inappropriate [(3), pp. 10, 26]. Concepts such as “out-of-frame” or “frame breaks” highlight occurrences that do not fit the expected frame and can cause confusion or encourage reframing of the situation. Out-of-frame events refer to actions or elements that do not fit the current context, breaking the norms of what is expected [(3), p. 201]. In contrast, frame breaks occur when an unexpected situation disrupts the established context, forcing participants to reconsider their operating framework [(3), p. 347]. Additionally, “Flooding out,” a concept discussed under frame breaks, refers to situations where an individual becomes overwhelmed by emotions or circumstances, making it difficult to maintain the expected frame. This overwhelming experience can lead to emotional flooding, where the person loses control over the situation, often breaking down or withdrawing [(3), pp. 350–359].

Guided by Goffman’s frame analysis (3), we aimed to identify overarching social frameworks in the Norwegian RC setting and examine how these frames influence social engagement and the sharing of experiences, knowledge, and skills.

### 2.7 Data analysis

The first author coded the transcripts of audio-recorded interviews and field notes using NVivo 15 software. All transcripts from observations, focus groups, and interviews were integrated into a single NVivo project for analysis, utilising the same analysis codebook for all materials. For this study, we conducted a qualitative content analysis with a directed approach (24), as initial codes from the first data collection and Goffman’s frame analysis (3) guided us in identifying key concepts as initial coding categories. Initially, we read transcripts and field notes line by line to familiarise ourselves with the data and identify framework-related topics. We noted themes such as aims, leading principles, information, norms and rules, competence, seating arrangement, and pedagogical means. We iteratively analysed the data, consistently referring to theoretical frameworks to identify social frameworks, out-of-frame events, and frame-break events. We also identified key concepts and keying strategies that guided students in deciding whether to share or withhold information. The authorship team collaboratively generated and discussed various mappings of the relationships between these emergent themes. Multiple iterations of theme maps were developed until the authors reached a consensus on the final representation.

## 3 Results

**Figure 1:**
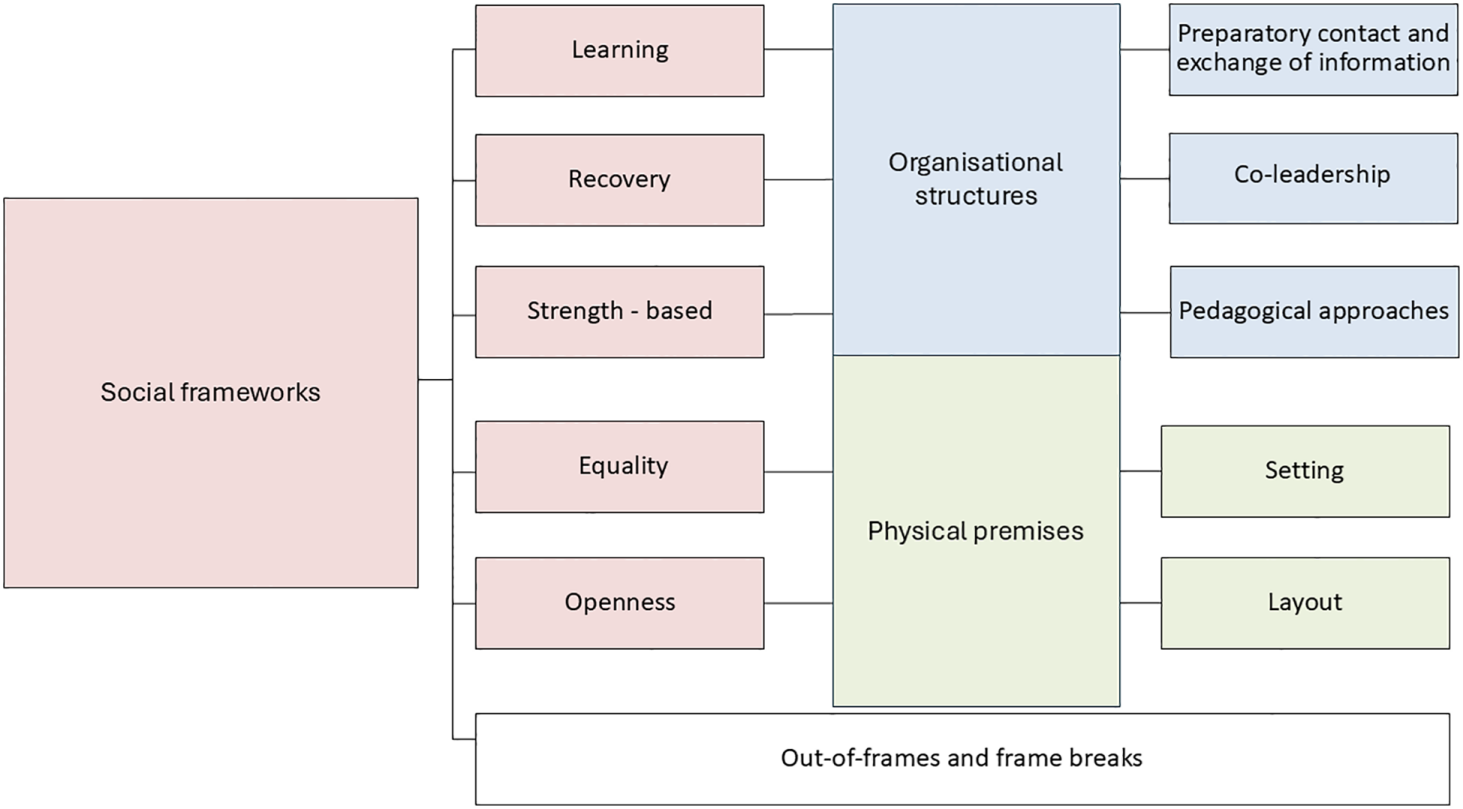
Analysis of social frames. illustrates the social frameworks and the elements, such as organisational structures and physical premises, that sustain these frameworks.

**Figure 2:**
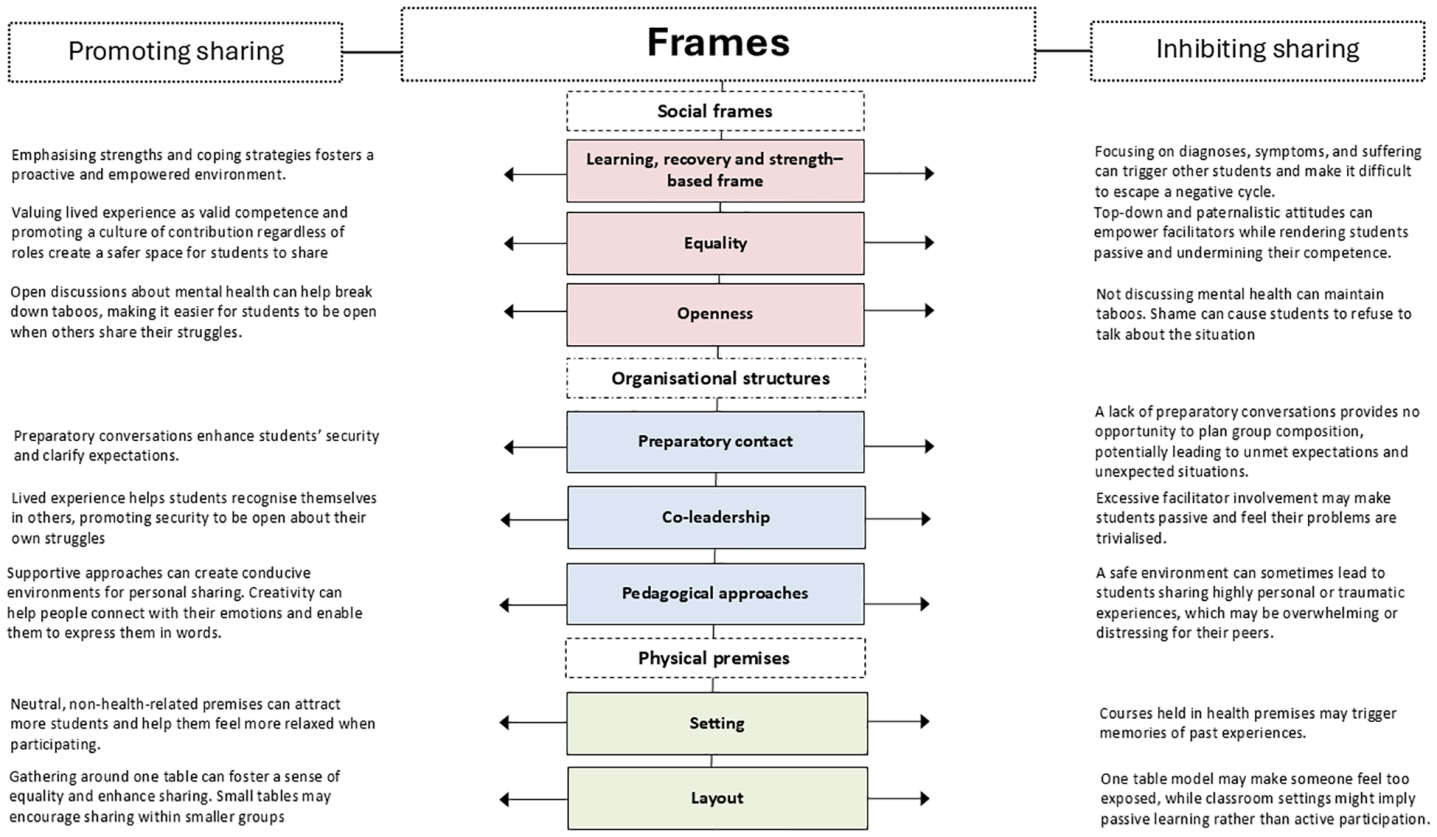
Frames promoting and inhibiting sharing. shows how organisational structures and physical premises promote or inhibit sharing. The dotted lines indicate that the social frameworks are organic. The challenge of categorising promoters and inhibitors into fixed boxes arises because they vary according to context and student preferences.

We conducted a qualitative study to explore how social frameworks inhibit and promote sharing in Norwegian RCs. Inspired by Goffman’s frame analysis (3), we identified (i) social frameworks and two aspects of the framework: (ii) organisational structures and (iii) physical premises (**Figure 1**). Our analysis revealed how various aspects of social frameworks across the two sites could influence students’ engagement and sharing (**Figure 2**).

### 3.1 Social frameworks

Our analysis identified five critical frames within the social framework, present at both sites: learning, recovery, strength-based, equality, and openness. Furthermore, we noted disruptions to the social framework.

Norwegian RCs’ primary aim is to shift the focus from a health to *a learning frame*, akin to international RCs. Facilitators emphasise that their courses are not treatment or targeting specific diagnoses but supplements to existing substance use and mental health services, concentrating on strengths and coping strategies rather than on diagnosis, symptoms, and suffering. A facilitator with experiential knowledge emphasised this significant shift in mindset:

Recovery is not about getting well but having a good life, even with challenges. Previously, one had to be healthy enough to work. Now, doing something meaningful, whether work or other activities, can lead to recovery. It is a change in perspective, and the gold in the philosophy is that no one is too sick to have a decent life.

The primary goal is to raise awareness of the students’ recovery process and equip them with knowledge and resources to lead fulfilling lives. While the courses do not offer direct treatment, they can still have therapeutic effects by fostering meaningful connections. A facilitator with experiential knowledge shared:

Attending a course for 5–7 weeks may not lead to a completely new life, but it can be a significant part of the recovery process. They might meet someone here and get new impulses that inspire new paths. In addition, it could have life-changing effects. Even if the course does not offer that, meeting others, gaining new insights, sharing experiences with others, and feeling that you are not alone in those challenges can have profound effects.

The RCs embody a *recovery frame*, emphasising the significance of gathering spaces that cultivate a sense of belonging (connectedness), mastery, self-discovery, and identity development. Students establish and pursue short-term goals as steps toward broader aspirations, frequently seeking community to mitigate feelings of isolation.

*The frame of equality* between facilitators and students and between professional knowledge and lived experience is emphasised. A facilitator with formal mental health training shared: “*Many [students] think that when they come to the course, we will teach [them] much stuff, but that is not how it is. We do everything together*.” Facilitators participate on equal terms with students; everyone is considered part of the group regardless of their role. Another facilitator with formal mental health training highlights the unique, inclusive space that RC provides:

…they have also provided a very inclusive space, which accommodates and, … lifts equality or evens out the differences that quickly otherwise prevail from my experience. So yes, that is why I keep being involved with the RC. Because I see that what happens there is unique.

Facilitators encourage *a frame of openness* about mental health to combat taboos. A facilitator with experiential knowledge emphasised the importance of discussing mental health openly to provide a nuanced and honest representation of living with such challenges. One facilitators with formal mental health training connect openness about feelings to the creative aspects of the courses:

Going to therapy and talking about feelings and things like that are not for everyone. Not everyone is as verbal; communicating [feelings] can be challenging, and you may not always feel connected. Creativity can often help to get in touch [with emotions] and to talk about the expression. When discussing creative expression, it is easier to start conversations about underlying challenges and talking about and feelings.

Facilitators often encounter scenarios where students’ expectations and behaviours deviate from the RC’s intended culture. New students, with limited understanding of promotional materials, might have misconceptions and expect traditional therapy instead of self-directed development. Facilitators noted that students often anticipate therapeutic outcomes misaligned with the RC objective:

There were expectations of a therapeutic effect and that they would get something that made them feel better … When I teach at a university or in a course elsewhere, no matter where, … people do not come with the expectation that they will have a personal recovery or that it will provide some therapeutic benefit. Moreover, I think there is a substantial difference, which contradicts what RC should be.

Students with extensive treatment histories might struggle to adjust to the RC’s self-work approach, and disruptive behaviours or unfiltered emotional expressions can create discomfort among students. During participant observations, we gained insight into such frame breaks. For instance, showing a movie portraying a girl’s transition from fear of living to fear of dying led to a sombre discussion where students shared their dark experiences, significantly changing the mood in the room. One student left the discussion and did not return to the course. Facilitators debated the appropriateness of discussing such experiences, balancing the need for openness against potential student distress.

Further, a poem on societal views of disabilities stirred strong emotions and a sense of injustice among students. Conflicts arose over the casual use of anxiety and depression terms, challenging the RC’s principles of equality and shared understanding. Students felt their struggles were trivialised when peers who did not have a diagnosis used these terms. A professional’s perspective that one cannot predict others’ experiences further provoked them.

Finally, initial non-participation created tension and insecurity for other students, but breakthroughs typically occurred when reluctant members began to share their personal experiences. These moments help establish trust and cohesion within the group, illustrating how deviations from expected behaviours impact group dynamics and sharing practices critical to the RC’s framework.

### 3.2 Organisational structures

We identified three organisational structures to maintain the social framework. These included preparatory contact and information exchange, co-leadership, and pedagogical approaches.

One crucial distinction between the sites was their contact level with students before the course. Site 1 engages in *preparatory conversations* with students before the course commencement. These discussions offer essential information, evaluate motivation, and establish clear expectations to reduce anxiety. Students tour the facility, meet the facilitators, and learn about the course themes related to the CHIME. Facilitators clarify that sharing involves reflecting on and setting personal goals related to these themes, which they then share with the group. One facilitator with experiential knowledge emphasised the voluntary nature of sharing personal experiences and the importance for students to set boundaries:

We discuss sharing what you feel comfortable sharing. You do not need to think that you must reveal your whole life story or all the negative experiences. When we present the course, we emphasise that it focuses on the present moment, and our approach is that we do not need to delve into the past so much or revisit painful topics. Although we address complex issues, it is a positively charged course.

A facilitator with experiential knowledge also emphasised the necessity of clarifying what sharing entails, as interpretations can vary among students. Conversely, another facilitator noted that as the course progresses, the concept of sharing becomes clearer through practical tasks. Facilitators avoid reviewing medical records before or during the course, and those who lead courses in the treatment system only document attendance and information that the students wish to be recorded. A facilitator with formal mental health training explains the reasoning:

*It is more important to document that they have completed the course, whether they attended or not. However, I do not read journals and communicate this to them because it is irrelevant to a recovery course. What diagnoses do they have, and what challenges have they faced? We prefer to focus on different aspects.*

This approach ensures that diagnoses do not sway participation and fosters a non-judgmental environment.

At Site 2, preparatory conversations are optional and can be requested by the students, focusing on normalising participation and ensuring a non-treatment-focused experience. Additionally, leaders of the RC send welcoming texts before each session to encourage attendance. One facilitator with formal mental health training elaborates on the background: “We wanted easy access to our courses. You should not get the experience of applying for service because we are not a health service; it is more like you enrolling in adult education, where preliminary conversations are not expected.” Registration involves minimal information, and facilitators meet students for the first time on the day of the course start. This approach prioritises meeting individuals as they are rather than focusing on their history. A facilitator with experiential knowledge elaborates: “We should meet people, not diagnoses, faults, or shortcomings (….). It is about meeting and getting to know the person where he is today, not where he was three years ago.” Facilitators often avoid asking questions and discussing diagnoses, but they frequently encounter students who bring it up and share information about their background. The facilitators who did not have prior contact with the participants provided information at the beginning to clarify that the courses do not offer therapy but rather provide increased knowledge and learning. This approach helps students strike a balance in their sharing and avoid delving into overly painful experiences. Sharing involves reflecting on assignments and discussing strategies for managing everyday life. Creative courses encourage the sharing of personal experiences through tangible outputs, such as paintings or photographs, making it easier to discuss emotions and challenges.

Facilitators and students have noted the importance of *co-leadership* between facilitators with formal mental health training and experiential knowledge. This combination enables the integration of theoretical concepts with real-life situations, making them more comprehensible and relevant. Facilitators with formal mental health training can elucidate psychoeducation and theories that help students understand human interactions and reactions. Meanwhile, peer support workers excel at focusing on the experiential aspects of situations, providing valuable insight and deeper understanding. This helps students engage with the material, fostering an open dialogue in which they feel comfortable sharing experiences and asking questions. A facilitator with formal mental health training stated:

Experiential knowledge contributes something to the course that we can only partially achieve as professionals. Furthermore, professionals who adopt a recovery-oriented approach reveal a human side that becomes quite evident to the course students and is somewhat difficult to articulate. There is an atmosphere in the room, an occurrence that is difficult to put into words. Nevertheless, it fosters a sense of security and openness. From my experience with all the courses I have conducted, a strong sense of security is established quickly within the course. This is due to the open sharing we practice.

Facilitators observed that sharing lived experiences made students feel safer participating and added a motivational element. The ability of facilitators with experiential knowledge to overcome challenges and return to everyday life is a significant source of motivation for students. Their unique perspectives and methods of knowledge transfer often exceed what professionals with solely formal training can offer. One facilitator with formal mental health training described a particular event:

When we presented “My Tree of Life”, it was evident that those invited were highly tense. With their jackets on and arms crossed, they sat silently until one of the peer support workers shared how she had designed her tree and the challenges she had faced. This completely transformed the mood in the room. There is something about the students knowing that they are encountering understanding—someone who has experienced this—that helps facilitate the course.

Facilitators with formal mental health training expressed that their course was not manual-based or subject-led, but rather grounded in facilitators and students sharing their personal experiences. The critical aspect, therefore, is personal identity over professional roles and titles. The role diverges from traditional educational settings, with facilitators often engaging alongside students and promoting active participation over passive learning. One facilitator with formal mental health training describes how this shapes how they present themselves to students:

*When I introduce myself, I first and foremost introduce myself as a mother and foster mother, and then, in a way, the title is further back, but I become more of a human being than hiding behind a title …* Young people often feel that it is harmless, and I am not a psychologist, doctor, or expert. Two people might understand them differently, even though I emphasise that I am professionally educated. However, that is not the first thing I say. I use other words first.

Facilitating a safe environment is crucial to the *pedagogical approaches*. Ground rules are co-created at the start of each course to ensure a respectful and secure learning environment that fosters sharing. Essential rules include moral confidentiality, mutual respect and respect for each other’s boundaries. Students affirmed that their ownership of how things should function in the classroom enhances group safety and encourages open sharing. Facilitators maintain these rules, reinforcing them when necessary to handle disruptions or unexpected behaviours. This was observed in the courses, where they referred to the ground rules when events deviated from the agreed framework, such as students dominating the group or displaying unexpected behaviour like hugging other students. In extraordinary situations, facilitators could contact managers to maintain a conducive environment for sharing.

Facilitating RC courses involves speaking calmly to ensure everyone can follow, using straightforward and understandable language, and coordinating as facilitators. They prepare more precisely what they would teach than in other teaching settings, considering that some students might be on medication, which could affect their learning. Such an approach can help students feel more comfortable sharing.

Facilitators described RC courses as dynamic and engaging, combining teaching with meaningful student learning. One facilitator with formal mental health training noted that RC students often struggle more than those in other educational institutions, requiring extra care while fostering independence.

RCs use *structured activities* tailored to the course’s themes and target groups to promote personal sharing. Activities included illustration cards, network mapping, and categorising personal values. Inspired by narrative therapy, the “My Tree of Life” encouraged students to represent different aspects of their lives. Activities such as “Five Chairs”, which explores various social behaviours, alongside “The Helping Hand”, which provides practical tools for understanding and handling thoughts and feelings, were also utilised. The lotus flower model, a structuring idea development approach, was used to help students acquire new strategies. Creative methods, such as metaphors, collage-making, and painting, facilitated self-expression.

### 3.3 Physical premises

Two physical premises were identified to support the social frameworks, including the setting and layout.

There were crucial distinctions within the physical premises between the sites, where Site 1 is situated adjacent to other mental health services, while Site 2 operates independently of these services. At Site 1, ongoing discussions are underway about relocating RC courses to non-health-related, neutral spaces. Conducting courses in neutral locations can help reach a broader range of students and make them feel more at ease when participating. One facilitator with experiential knowledge described that courses held in health buildings may adversely affect some students due to past experiences: “You might tense up a bit when you’re about to enter a health facility … It’s related to some experience you’ve had with a health facility. You might get a different reaction if you frame it in a neutral place that isn’t called a health facility.” However, another facilitator mentioned that health buildings are easily accessible, making them a convenient option for those who do not have permanent premises independent of other health or social services. The options range from municipal buildings, such as libraries or premises related to substance use and mental health services, to others within health buildings.

The physical *layout* varies based on the needs of individual courses. One model involves gathering everyone around a single table, regardless of their roles, to promote equality and foster a sense of group identity between facilitators and students. Then, facilitators conduct the course at the same level as the students. Some facilitators elaborate on the reasoning:

…if we were to remove the distinction between experience and subject, it was essential to remove the distinction between course facilitators and students. There was something about being on an equal footing with creating social security in that setting (facilitator with formal mental health training).

Most of the magic in recovery courses lies in mirroring when someone shares something, and you recognise it within yourself. Then you need that closeness; you cannot sit too far apart. You must be able to share across the table among the different students (facilitator with experiential knowledge).

Thus, the idea is that such a layout would make it safer for the students. Facilitators believed that the experience of equality would be more significant if they did not stand in front of the students and teach. In this model, the facilitators used flipcharts only when they needed to write collaboratively, such as defining what “hope” meant to the group. Facilitators with formal mental health training believed that this way of organising the room was more equal than traditional classroom setups, where there is often more one-way communication.

Another model involved a sofa model where everyone sits together on sofas. A facilitator with experiential knowledge conveyed that this setup created a more relaxed atmosphere than tables and chairs. She found that students seemed more comfortable when they had blankets and pillows and could take their legs up on the sofa.

A different model involved arranging students around small tables, with approximately four students seated at each table. Facilitators stood at the front of the room, using PowerPoint and flipcharts, and moved around to each group during structured activities. Generally, students choose where they want to sit. If students leave their name tags and course booklets, facilitators place them on a neutral table, and then students sit where they prefer. Some students can become stressed if they cannot sit in the same place they previously did. When facilitators know the group well, they sometimes assign seating by placing name tags to control where individuals should sit.

In contrast, in a creative course, students initially sat in a circle without a table at the beginning and end of the course. A facilitator with formal mental health training highlighted that several students, including those with professional knowledge, experienced this as unsafe. However, evaluations conducted by the RCs after the courses showed that many students gave positive feedback, particularly valuing the sharing and feedback within this group setup. Additionally, one student noted that small tables fostered a sense of safety as she got to know a small group at a time before rotating and eventually familiarising herself with the whole group. She contrasted this experience with another course where the seating arrangement around a single table felt less safe, making her feel overly visible and exposed. Some facilitators expressed in the courses that students could leave the room if they felt overwhelmed, accompanied by a facilitator. This gives the students a sense of security and control over their participation.

## 4 Discussion

Our study addresses the fundamental purpose of exploring social frameworks, focusing on how organisational and physical elements influence sharing among students and facilitators in Norwegian RCs. Specifically, we structured our inquiry around the question: *How do social frames promote and inhibit sharing in Norwegian Recovery Colleges?*

Previous research has provided valuable insights into the guiding principles of RCs, emphasising elements such as equality, co-creation, learning, social connectedness and recovery-oriented approaches (1, 2, 5). These principles advocate for integrating experiential, clinical, and theoretical knowledge, enabling co-leadership, co-creation of knowledge and mutual learning in a diverse educational setting. Studies on students’ perspectives have shown that co-leadership and learning in diverse educational settings are particularly beneficial (12), alongside voluntary participation, relevant content and practical tools they can implement in their everyday lives (14). At the same time, previous research has demonstrated how the course elements and relationships between students and facilitators may contribute to dropout (6, 19).

By incorporating the lens of Goffman’s frame analysis (3), our study adds a new layer of understanding to the cultural and structural aspects of RCs. We identified critical social frameworks within RCs encompassing learning, recovery, strength-based, equality and openness. Additionally, organisational structures involving preparatory conversations and information exchange, co-leadership, and approaches played a pivotal role in sustaining these frameworks. Physical premises, including the local and the organisation of the spaces, further influence these frameworks (**Figure 1**).

Our empirical data underscores the significance of these elements in promoting sharing practices. As Goffman [(3), p. 247] states: “The organisational premises – sustained both in mind and in activity – I call the frame of the activity.” This quote illustrates the critical role that organisational structures and physical premises play in shaping and supporting the social frameworks RCs aim to build. Integrating these elements creates a comprehensive environment that facilitates sharing practices between students and facilitators.

Our results demonstrate that social frameworks, including their organisational structures and physical premises, are vital in promoting sharing. These aspects, designed to foster a supportive and empowering environment, can occasionally both promote and inhibit sharing, acting as a double-edged sword. The social frameworks are dynamic and vary based on the context, the facilitators, and the student group in each course (**Figure 2**). The organic aspect of the social frames serves as a theoretical contribution to understanding the complexities and contextual variations in RC environments.

The social frameworks are centred on the RC’s ideology of personal and social recovery principles (1, 2, 5). This ideology is claimed to foster an empowering environment by shifting the focus from therapy to learning, and valuing strengths and coping mechanisms rather than diagnoses (2, 5). Acknowledging lived experience as a valid form of competence makes students feel that their contributions are significant. Facilitators promote a culture of sharing based on equality, where everyone, regardless of their role, shares their experiences, creating a safer space for dialogue (10, 25). Open discussions about mental health help dismantle taboos and reduce stigma (17, 26), making students more comfortable sharing their struggles.

Interpreting social frameworks can be challenging. Both students and facilitators may bring elements from traditional service models, which can disrupt the social framework RCs seek to establish. Successful sharing relies on facilitators and students similarly interpreting and negotiating these frames to foster a mutual understanding of expectations and surroundings. Different interpretations of these frames can lead to mismatched expectations and “frame breaks” (3). Such frame breaks can manifest as oversharing, limited sharing, emotional breakdowns, or excessive facilitator involvement. According to Goffman [(3), p. 380], interaction settings such as RCs are designed to engage participants deeply, encouraging active participation due to their therapeutic effects (26). While the intensity may overwhelm some, it often stems from effective management rather than inappropriate leadership (3).

The organisational structures are crucial in shaping how students interpret and engage in activities, guiding their behaviour toward sharing (3). This involves exchanging information that forms expectations, emphasising lived experience, and pedagogical approaches. Data indicate no explicit rules or consistent conventions regarding what students should share and what they should not. A lack of explicit rules or conventions can hinder sharing for those who feel uncertain about what is appropriate, leading to excessive sharing or overwhelming feelings. This variability highlights the importance of personal comfort and individual boundaries, with some facilitators advising against discussing personal traumas, while others leave the topic more open-ended.

Preparatory conversations enhance initial engagement, improve students’ security, clarify expectations, and mitigate potential “frame breaks”. Conversely, a lack of regular preparatory conversations can lead to mismatched expectations, which, according to Thériault et al. (6), may result in student dropout. Additionally, it reduces initial engagement and missed opportunities to plan group composition effectively. The facilitators who were obligated to document information from the courses in the student’s medical file only documented what the students requested. This practice ensured a non-judgmental environment, encouraging open participation without fear of information being reported. The reasoning behind not conducting preparatory conversations is related to avoiding associations with therapy, which often includes medical records. This approach could promote sharing for some, showing that what we highlight as promoters of inhibitors does not necessarily function as such.

Experiential knowledge is a facilitating element in the courses (6, 17) and employing facilitators with lived experience enhances students’ sense of security. Sharing personal stories helps students see themselves in others, boosting their confidence in being open about their issues [also found by (26)]. Facilitators with formal mental health training who also share their personal experiences can dismantle hierarchical dynamics, fostering greater sharing. The facilitator’s involvement in the sharing practice can operate as a “disruption for” the frame, promoting sharing from the other students (10). Field notes also reflect that excessive involvement from the facilitator may sometimes lead to student passivity or the trivialisation of their problems, disrupting the social framework and breaking down interaction and social ties [(27), p. 366] across roles. For instance, two students felt their struggles were trivialised when peers without a diagnosis used specific terms too casually. Such situations challenge the social frame of equality and sharing in RCs, where facilitators share their struggles with the students.

According to Goffman’s frame analysis (3), events such as RC generate a “social world” where individuals may take on opposing positions, perhaps due to differing goals, perspectives, or roles. Understanding guided doings involves acknowledging physical control and the social interactions, including conflicts or negotiations, that are part of the event. Opposing positions contradict their efforts towards equality, and what appears to be a blurring of roles. Facilitators and students with formal mental health training have different motivations for participating in the course than students in recovery, which means that different frameworks apply according to their respective positions. They have responsibilities for the students, assess their position in the room, and determine what is appropriate to share to promote the desired sharing from students [as elaborated in (10)]. We may consider these opposing positions as “real” and “pseudo” students. Despite facilitators’ efforts to dismantle hierarchy and promote equality (25), the distinction between those with lived experience and professional backgrounds often becomes evident (10). This dynamic raises essential questions about the impact of professionals sharing their experiences and the potential feelings of shame or frustration among students. Understanding these dynamics is crucial for tailoring RC environments that foster effective sharing and mutual support. This aligns with Andersen et al. (19), who noted that students with formal mental health training might feel alienated in the course when they lack relevant personal experiences to share. Our study indicates that students with substance use and mental health challenges might feel alienated when those with a professional background share something that disrupts the sharing flow. Likewise, Al-Adili et al. (17) found that facilitators who spent too much time on their own experiences created barriers in the course. When their contributions were out of touch with the RC principles, they were seen as disturbing. This discussion illustrates that the professional sharing dynamic can inadvertently alienate some students.

Supportive approaches can create conducive environments for personal sharing. This includes co-creating ground rules, using clear and understandable language, and providing additional student support. The RCs facilitated structured activities to promote sharing. Activities include illustration cards, network mapping, categorising personal values, and “My Tree of Life”, offering varied and creative avenues for reflection, self-expression, and personal storytelling. The Five Chairs approach (28), the lotus flower model [(29), p. 300], and The Helping Hand from Psychological First Aid (30, 31) provide practical tools to raise awareness about behaviours and find helpful solutions. Preliminary personal assignments and reflection time helped students connect their experiences and fostered sharing within the group.

Nurser et al. (26) research highlights how feeling safe facilitates richer disclosure; however, this increased sense of safety can sometimes lead to oversharing, where highly personal or traumatic disclosures may overwhelm or distress peers. Some students’ oversharing may inhibit others from sharing. Andersen et al. (19) also note this phenomenon, stating that specific relational dynamics can influence participant engagement and lead to dropouts. We have illustrated instances where the focus shifts to diagnoses and suffering that can disrupt the social framework of strengths-based discourse norms (2, 5). These shifts represent an example of what Goffman (3) refers to as out-of-frame situations that do not fit the context. They can dominate group discussions and trigger negative cycles, leading to frame breaks (3). For example, a student’s withdrawal after another’s trauma disclosure and oversharing exemplifies Goffman’s concept of “flooding out” and “frame break” [(3), pp. 380–381]. This illustrates that supportive approaches do not necessarily promote the sharing desired in RCs.

On the other hand, limited sharing disrupts the social frame of mutual learning in the course. A notable turning point in one group occurred when a previously reluctant student broke down in tears after her disclosure. According to Goffman, such intense situations can increase emotional involvement and cohesion among students, fostering in-depth conversations and personal development [(3), pp. 380–381]. This breakdown illustrated the potential effectiveness of frame breaks, as her honesty led to genuine involvement from others, who then offered support by giving hugs and a pat on the shoulder and shared similar experiences. Tavory and Fine [(27), pp. 366–367)] refer to this type of frame break as “disruption for”, which involves entering the social framework, potentially giving rise to new, deeper modes of intersubjectivity and social coordination. According to Goffman (3), the emotional intensity of these moments can foster engagement and empathy among students, creating a supportive environment where more individuals feel encouraged to share their thoughts and feelings. This mutual support can achieve connectedness among individuals who have faced similar situations. Dialogue following a frame break can help students find meaning in difficult situations, while sharing personal strategies can promote hope and empowerment. Through such experiences, individuals can strengthen their identities as they gain a deeper understanding of and acceptance for themselves and their challenges.

The physical premises are visible keys that guide students towards expected behaviour and contributions in sharing. Courses are held on various premises, potentially evoking associations with other mental health services, which may inhibit sharing. Conversely, permanent premises possibly create a more stable and comfortable environment. RCs prefer neutral, non-health-related premises for their courses (5), helping students feel more relaxed when participating. Not all RCs have access to these premises, which may trigger memories of past experiences and inhibit sharing. RCs organise the physical layout to foster equality and enhance sharing, but some setups may feel too exposed for some students. Co-location with other services can work well for students who are also in contact with these services, but this may be challenging for others who have progressed further in their recovery. Regardless, co-location will make it difficult for students to perceive this as a distinct new service from the regular offerings, which can affect their understanding of the intended sharing practice.

The RCs apply various room arrangements. A one-table model fostered a sense of equality and direct communication, facilitating open sharing in plenary sessions. This setup fostered unity and inclusiveness by enabling all participants to interact directly with one another. However, the one-table model could also feel too exposed for some students, potentially inhibiting open sharing for those less comfortable in a larger group setting. Small table arrangements dominated, with facilitators standing in front and moving between the different groups. This arrangement promoted intimate group discussions and personalised interactions within smaller groups, but it also limited plenary contributions and cross-group sharing. Such an environment could encourage a safer environment for shy or less confident students, but might inhibit broader group engagement and the sharing of diverse perspectives. The presence of tables, chairs, canvases, course booklets, and the facilitator moving between the groups creates a structured environment for teaching and learning. While small tables may promote sharing within smaller groups, a setup associated with a classroom setting can potentially convey passive learning rather than active participation.

### 4.1 Implications for practice

Based on the study’s findings, several implications can influence students’ contributions to sharing in the RC setting. Given the organic nature of course interactions, the factors that promote or inhibit sharing are contextual. We have indicated that organisational structures and physical premises impact information sharing.

A social framework that emphasises valuing strengths, lived experiences, and open discussions about mental health encourages sharing, while emphasising diagnoses and top-down attitudes may inhibit it. Successful sharing relies on facilitators and students similarly interpreting and negotiating social frameworks to foster mutual understanding and expectations.

Organisational structures within this framework, such as clear communication, preparatory conversations, and respect for boundaries, foster sharing, whereas a lack of preparation and excessive facilitator involvement can hinder it. Physical premises, such as neutral environments, may enhance sharing, while health-related settings and overly exposing arrangements can obstruct openness.

Facilitators must balance the physical setup and pedagogical approaches to ensure it is safe and comfortable enough to encourage sharing, yet dynamic enough to challenge students and promote new learning. For example, enabling students to change seats and interact with different peers at each session can foster engagement and break the monotony of fixed group interactions, tailoring the setting to accommodate each student’s varying comfort levels and needs.

### 4.2 Limitations

In this article, we have emphasised the leader and facilitator perspective, while examining students’ perspectives to a lesser extent. Although our focus has provided valuable insights, it also means that the voices and experiences of students have not been as prominently featured. The article is part of a larger study, where other articles have thoroughly addressed the student perspective. Nonetheless, integrating the students’ and facilitators’ perspectives more evenly in this study could have offered a more nuanced understanding of the framing and functioning of RCs.

Explicit comparisons between the two sites could have provided valuable insights into their operational frameworks. While we have strived to safeguard the anonymity of participants by not disclosing excessive information about the data’s origin, this may limit the depth of contextual analysis. Finally, each RC operates within its unique local environments, which may influence the generalisation of how social frameworks promote or inhibit sharing within other contexts.

Based on these limitations, we recommend that future research include a broader and more diverse sample of study participants to better represent the spectrum of experiences and understanding of social frameworks found in RCs.

## 5 Conclusion

This article illustrates how RCs consciously frame sharing to achieve mutual learning among the students. These social frameworks involve organisational structures and physical premises that influence students’ behaviour and guide them towards sharing. Our study highlights the organic and contextual nature of these frameworks. What promotes sharing in one setting may act as an inhibitor in another, demonstrating the complexity of these dynamics. Recognising this complexity is crucial for facilitators in RCs.

## Data Availability

The datasets for this article are not publicly available due to concerns regarding participant anonymity. Requests to access the datasets should be directed to the corresponding author.
